# Respiratory Pathways Reconstructed by Multi-Omics Analysis in *Melioribacter roseus*, Residing in a Deep Thermal Aquifer of the West-Siberian Megabasin

**DOI:** 10.3389/fmicb.2017.01228

**Published:** 2017-06-30

**Authors:** Sergey Gavrilov, Olga Podosokorskaya, Dmitry Alexeev, Alexander Merkel, Maria Khomyakova, Maria Muntyan, Ilya Altukhov, Ivan Butenko, Elizaveta Bonch-Osmolovskaya, Vadim Govorun, Ilya Kublanov

**Affiliations:** ^1^Winogradsky Institute of Microbiology, Research Center of Biotechnology, Russian Academy of SciencesMoscow, Russia; ^2^Saint Petersburg State University of Information Technologies, Mechanics and OpticsSt. Petersburg, Russia; ^3^Belozersky Institute of Physico-Chemical Biology, Lomonosov Moscow State UniversityMoscow, Russia; ^4^Federal Research and Clinical Centre of Physico-Chemical MedicineMoscow, Russia; ^5^Moscow Institute of Physics and TechnologyDolgoprudny, Russia; ^6^Laboratory of Microbial Genomics, Immanuel Kant Baltic Federal UniversityKaliningrad, Russia

**Keywords:** deep subsurface environment, West-Siberian megabasin, thermophilic bacteria, respiratory metabolism, cytochrome oxidases, arsenate reductase

## Abstract

*Melioribacter roseus*, a representative of recently proposed Ignavibacteriae phylum, is a metabolically versatile thermophilic bacterium, inhabiting subsurface biosphere of the West-Siberian megabasin and capable of growing on various substrates and electron acceptors. Genomic analysis followed by inhibitor studies and membrane potential measurements of aerobically grown *M. roseus* cells revealed the activity of aerobic respiratory electron transfer chain comprised of respiratory complexes I and IV, and an alternative complex III. Phylogeny reconstruction revealed that oxygen reductases belonged to atypical *cc(o/b)o_3_*-type and canonical *cbb_3_*–type cytochrome oxidases. Also, two molybdoenzymes of *M. roseus* were affiliated either with Ttr or Psr/Phs clades, but not with typical respiratory arsenate reductases of the Arr clade. Expression profiling, both at transcripts and protein level, allowed us to assign the role of the terminal respiratory oxidase under atmospheric oxygen concentration for the *cc(o/b)o_3_* cytochrome oxidase, previously proposed to serve for oxygen detoxification only. Transcriptomic analysis revealed the involvement of both molybdoenzymes of *M. roseus* in As(V) respiration, yet differences in the genomic context of their gene clusters allow to hypothesize about their distinct roles in arsenate metabolism with the ‘Psr/Phs’-type molybdoenzyme being the most probable candidate respiratory arsenate reductase. Basing on multi-omics data, the pathways for aerobic and arsenate respiration were proposed. Our results start to bridge the vigorously increasing gap between homology-based predictions and experimentally verified metabolic processes, what is especially important for understudied microorganisms of novel lineages from deep subsurface environments of Eurasia, which remained separated from the rest of the biosphere for several geological periods.

## Introduction

Recently the discovery and study of *Ignavibacterium album* and *Melioribacter roseus* (Iino et al., 2010; Podosokorskaya et al., 2013), the first two cultivated representatives of previously uncultured candidate division ZB1 (Elshahed et al., 2003) led to the proposal of Ignavibacteriae phylum, a member of the Chlorobi-Bacteroidetes-Ignavibacteriae group (Podosokorskaya et al., 2013). Besides two cultured species Ignavibacteriae includes numerous clones found in various environments all around the world, including hot springs, oil reservoirs, mines, and seafloor sediments (Tiodjio et al., 2014; Kato et al., 2015). *Melioribacter roseus* was isolated from a microbial mat proliferating in a geothermal water discharge (Podosokorskaya et al., 2013). The organism is a moderate thermophile and originates from subsurface biosphere, as 16S rRNA genes of closely related organisms were identified in arsenic-containing water samples from 2725 m depth at the same site (Frank Y. A. et al., 2016).

Physiological studies revealed that both cultured representatives of the phylum Ignavibacteriae are facultatively anaerobic organotrophs capable of fermentative growth on various carbohydrates and of oxygen or arsenate respiration with acetate as the electron donor. Primary genome analysis of *M. roseus* highlighted key determinants of electron transport chains, providing important insights into the organism’s ability to oxidize various electron donors during aerobic or anaerobic respiration. Genes for the electron transfer chain membrane complexes I and II, alternative complex III (ACIII) and several terminal oxidoreductases transferring electrons to oxygen and arsenate were found. Among those, the three different oxygen reductases and two different molybdopterin oxidoreductases have been proposed to determine most active respiratory processes performed by *M. roseus*—aerobic respiration and dissimilatory arsenate reduction, respectively (Kadnikov et al., 2013; Podosokorskaya et al., 2013), although their specific roles in respiratory metabolism were not assigned.

All the currently known energy-transducing oxygen reductases of respiratory chains in prokaryotes are subdivided into two large superfamilies. One of them, that encloses the enzymes with heme-copper binuclear center, is subdivided into three large clades known as A(I)-, B(II)-, and C(III)-type oxygen reductases (Mattar and Engelhard, 1997; Sousa et al., 2012). The other one, lacking copper, is represented by cytochrome *bd* oxygen reductases. With respect to the mode of energy transduction, the *bd*-type oxidases were shown to be the redox loops without ion-pumping activity (Borisov et al., 2011), while the heme-copper oxidases were demonstrated to pump protons (Wikström, 1977; Rauhamäki et al., 2012; Rauhamäki and Wikström, 2014) or sodium ions (Muntyan et al., 2015) across the cell membrane. In the respiratory chains of prokaryotic aerobes, the heme-copper oxygen reductases are highly diverse. Each of their clades, A, B and C, encloses enzymes accepting electrons from different donors, whether quinols or cytochromes *c*. As regarding the heme content, these enzymes can include hemes of *a*, *b*, *c*, and *o* types. However, molecular phylogeny of heme-copper oxidases superfamily reflects only one structural parameter affecting their catalytic activity. Namely, the position of conserved amino acid motifs that determine the structure of proton pumping channels is considered upon phylogenetic reconstructions (Sousa et al., 2012; Gennis, 2013). Recent multivariate analysis of genomic, structural, functional and thermodynamic information pertinent to the evolution of heme-copper oxidases has also highlighted correlation of their phylogeny-based grouping into three clades (A, B, and C) with the affinity to oxygen, and led to the proposal of the low O_2_-affinity A-type enzymes as the most recent evolutionary innovation and the high-affinity O_2_ reductases (B and C) arising from NO-reducing precursor enzymes (Ducluzeau et al., 2014).

Currently known respiratory arsenate reductases belong to the Arr family within a complex iron–sulfur molybdopterin oxidoreductase superfamily (CISM) — a group of molybdenum-containing enzymes, highly diverse by the catalyzed reaction. Among arsenate reductases only Arr-type enzymes are known to serve for energy generation during As(V) reduction to As(III), although functional flexibility of CISM superfamily enzymes could not rule out the presence of arsenate-reducing activity in the groups of molybdopterin oxidoreductases other than Arr (Duval et al., 2008; Rothery et al., 2008).

Here we describe the results of the phylogenetic analysis of putative oxygen and arsenate reductases genes in *M. roseus*, transcriptomic and proteomic experimental evaluation of their involvement in energy metabolism of the organism, and propose the mechanisms for aerobic respiration and arsenate reduction in this representative of the novel bacterial phylum. This information may cast light on yet poorly understood metabolic processes, occurred in thermophilic microbial communities residing in deep subsurface.

## Materials and Methods

### Cultivation of *M. roseus*

*Melioribacter roseus* P3M-2^T^ was incubated at 52–54°C in the modified anaerobic S medium (Podosokorskaya et al., 2013). Sodium sulfide and resazurin were only added to a strictly anaerobic variant of the medium for fermentative growth testing (see below). In all the cases of anaerobic growth, the strain was incubated in the dark without shaking. For aerobic or microaerobic cultivation, the same medium was used, but in this case, boiling and flushing of the medium with nitrogen were omitted, and inoculated tubes or bottles were incubated with shaking. Oxygen level in aerobic and microaerobic cultures was determined at the initial point and at the end of incubation by gas chromatography as previously described (Frank Y. A. et al., 2016).

All tests were performed in the presence of 10 mM Tricine buffer and yeast extract added as a source of growth factors (0.1 g 1^-1^ under anaerobic conditions and 0.05 g 1^-1^ under aerobic conditions).

### Inhibitory Analysis

Two inhibitors of electron transfer—rotenone and 2-*n*-heptyl-4-hydroxyquinoline-*N*-oxide (HQNO)—were used for the analysis. The inhibitors (each by 15 μM) were added to the medium from anaerobic (N_2_) stock solutions in 100% DMSO before cultivation. Negative control without inhibitors and additional positive controls, containing cells, acetate or maltose, air in the gas phase, and 0.1% (v/v) DMSO but no inhibitors, were used to provide data normalized to 100% activity to help estimate the impact of the inhibitors. Hydrogen was added to the gas phase (1 gauge atmosphere) to eliminate the possibility of fermentative growth of the strain on yeast extract (added to the medium as a growth factor at a concentration of 0.1 g 1^-1^).

### Transmembrane Electrical Potential and Respiratory Activity

The transmembrane electrical potential (Δψ) of the bacterial cells grown under atmospheric oxygen was detected using tetraphenylphosphonium (TPP^+^) penetrating cation at the final concentration of 1.6 μM and a TPP^+^-selective electrode, as described earlier (Kamo et al., 1979) with our modifications (Muntyan et al., 2012). The experiments were conducted with washed resting cell suspensions (3 mg ml^-1^ of total cell protein), prepared in fresh sterile aerobic culture medium (pH 7.0) lacking organic growth factors, in a conical thermostated glass cell at 52°C under vigorous aeration with a magnetic stirring bar. To evaluate the respiratory activity of bacterial cells grown under atmospheric oxygen, the rate of oxygen consumption was measured in the cell suspensions using a standard oxygen Clark-type electrode and a polarograph LP7e (LP, Czech Republic) at 52°C. All the measurements were performed in three biological replicas, i.e., with three different cell suspensions. Each cell suspension was divided into halves for simultaneous determination of Δψ and respiratory activity.

### Sequence Analysis

Phylogenetic analysis of CISM proteins was performed in the same manner as described by Sorokin et al., 2016. Phylogenetic analysis of cytochrome oxidases was performed as follows: all 725 seed sequences of Pfam PF00115 (COX1) family were downloaded from http://pfam.xfam.org/family/PF00115\#tabview=tab3. The sequences were clustered based on 50% identity threshold using CD-hit (Huang et al., 2010), and 1 representative of each cluster was left (112 sequences). Upon addition of two *M. roseus* homologs (MROS_0038 and MROS_1513) to this 96-sequence dataset, the sequences were aligned in MAFFT v. 7 (Katoh et al., 2002). The Le Gascuel (+I + G) model was revealed by ProtTest 2.4 (Abascal et al., 2005) to give the highest likelihood. Phylogenetic analysis was performed in MEGA v. 6 (Tamura et al., 2013).

Localization of molybdoenzymes was predicted basing on the results of six different on-line prediction services – SignalP 4.1, TatP 1.0, SecretomeP 2.0a and TMHMM 2.0 (all at CBS Prediction Servers^1^), as well as PSORTb 3.0.2^2^ and Phobius^3^.

### Membrane Fraction Preparation

For preparation of membrane fractions for proteomic analysis, *M. roseus* was grown fermentatively with maltose on a strictly anaerobic (*E_h_* -130 mV vs. standard hydrogen electrode [SHE]) medium and by aerobic respiration (atmospheric oxygen) with acetate in three biological replicas, 2 liters each. Growth was controlled microscopically, and the cells were collected on the boundary of exponential and stationary growth phases by centrifugation at 14 000 *g*, 4°C for 15 min. Cell pellets were washed in 50 mM Tris-HCl, pH 7.5 and centrifuged at 14 000 *g*, 4°C for 20 min. After that, cell pellets were resuspended in 50 mM Tris-HCl, pH 7.5 with 0.1 mg ml^-1^ DNAse I (Fermentas) and protease inhibitor cocktail (prepared from Protease Inhibitor Tablet, Sigma, according to manufacturer’s recommendations), and then they were sonicated on ice using Soniprep 150 Plus disintegrator (MSE, UK) for 5 min at a frequency of 12 kHz. Cell debris was separated by centrifugation at 14 000 *g*, 4°C for 8 min and discarded, while the supernatants were further ultracentrifuged at 100 000 *g*, 4°C for 1 h. Precipitated membrane fractions were stored at –80°C before analysis. Each fraction contained ca. 200 μg total cell protein as determined by the Bradford Protein Assay Kit (BioRad) and according to the manufacturer’s recommendations.

### Protein Extraction and Trypsin Digestion

The membrane fractions were treated with 5 μl of 10% RapiGest SF (Waters) and 1 μl nuclease mix (GE Healthcare) for 30 min at 4°C, resuspended in 45 μl of 100 mM NH_4_HCO_3_, vortexed and heated at 100°C for 5 min. After cooling to room temperature, insoluble material was removed by centrifugation at 15 000 *g* for 5 min. The supernatant was separated, checked for protein concentration, subjected for disulfide bonds reduction with 10 mM 1,4-dithiothreitol (DTT, BioRad) in 100 mM ammonium bicarbonate at 60°C for 30 min, and subsequently alkylated with 30 mM iodoacetamide (BioRad) at room temperature in dark for 30 min. DTT and 100 mM ammonium bicarbonate were added iteratively. After that, alkylated trypsin (Trypsin Gold, Mass Spectrometry Grade, Promega) was added to the supernatant in the ratio of 1/50 (mg per mg total protein) and incubated at 37°C overnight. For trypsin inactivation and degradation of the acid-labile surfactant, an aliquot of trifluoroacetic acid (Sigma) was added to the final concentration of 0.5% (m/v), and the mixture was incubated at 37°C for 45 min. Residual surfactant was removed by centrifugation at 15 000 *g* for 10 min. The obtained hydrolysate was desalted using a Discovery DSC-18 Tube (Supelco) according to the manufacturer’s protocol. Peptides were eluted with 700 μL 75% acetonitrile (ACN), 0.1% trifluoroacetic acid (TFA), dried in a SpeedVac (Labconco) and resuspended in 3% ACN, 0.1% TFA to the final concentration of 5 μg μl^-1^.

### LC-MS/MS Analysis

The LC-MS/MS was performed on a TripleTOF 5600+ mass-spectrometer operating in a data-dependent mode with a NanoSpray III ion source (ABSciex, Canada) coupled to a NanoLC Ultra 2D+ nano-HPLC system (Eksigent) configured as described by Ziganshin et al. (2016).

### Protein Identification

The raw LC/MS-MS datasets (.wiff file format) were converted to Mascot Generic Format (.mgf file format) using AB SCIEX MS Data Converter (version 1.3). Proteins were identified with the Mascot search engine (version 2.5.1) against the *Melioribacter roseus* str. P3M sequence database (RefSeq: NC_018178, which contains 2840 amino acid sequences). The Mascot searches were performed with the following parameters: tryptic-specific peptides, maximum of one missed cleavages, a peptide charge state limited to 1+, 2+, and 3+, a peptide mass tolerance of 10 ppm, a fragment mass tolerance of 0.5 Da, and variable modifications caused by Oxidation(M) and Carbamidomethylation(C). The False Discovery Rate (FDR) was calculated using the decoy database analysis with Mascot. Individual ions score higher than 11 indicate identity or extensive homology with *p* < 0.05 and FDR <1%. The mass spectrometry proteomics data have been deposited to the ProteomeXchange Consortium (Vizcaíno et al., 2014) via the PRIDE partner repository with the dataset identifier PXD003662 (refer to additional information below for details).

### Quantitative Proteomics

For comparative analysis of protein amount, emPAIs (Ishihama et al., 2005) were calculated. Data were normalized using the scaling method. Proteins were considered to be statistically different according to the unpaired two-tailed Student’s *t*-test (*p*-value <0.05) with the Benjamini and Hochberg (1995) adjustment for *p*-values.

### Transcriptomic Analysis

For transcriptomic analysis, seven sets of primers for *M. roseus* genes encoding the catalytic subunits of three oxygen reductases (*coxI*, *ccoNO*, *cydA*) and two molybdopterin-containing oxidoreductases (*ttrA* and *psr/phsA*), as well as for the reference housekeeping genes *rpoB* (encoding the DNA-directed RNA polymerase subunit beta, MROS_0223) and *atpA* (encoding the alpha subunit of F0F1–type ATP-synthase, MROS_0272), were designed using the Primer-BLAST service (http://www.ncbi.nlm.nih.gov/tools/primer-blast/). The specificity of the primers was verified by the Sanger sequencing of amplicons. The primers are summarized in Supplementary Table S1. RT-PCR analysis was performed with the cells grown at five different cultivation conditions: aerobic respiratory growth at atmospheric O_2_ concentration in the gas phase, microaerobic respiratory growth at 2% O_2_ in the gas phase, anaerobic respiratory growth with arsenate, fermentative growth in the absence of external electron acceptors at positive *E_h_* value of the medium (+200 mV vs SHE) or negative *E_h_* (–130 mV vs SHE, corresponding to strict anaerobiosis achieved by addition of 1 mM sodium sulfide to the culture medium). At all three respiratory cultivation conditions, a non-fermentable substrate acetate was used, while at both fermentative cultivation conditions, the anaerobic media were amended with a fermentable substrate maltose. In all the cases, the total RNA was extracted using ExtractRNA and CleanRNA Standard kits followed by DNAse I treatment. To prepare cDNA, 2 μg of the total RNA was reverse-transcribed using the MMLV RT kit. All the chemicals at this stage were from Evrogen, Russia. Quantitative PCR (qPCR) was performed using the qPCRmix-HS SYBR kit (Evrogen, Russia) on a StepOnePlus^TM^ Real-Time PCR System (Applied Biosystems, United States). Calibration curves were constructed based on fivefold dilutions of genomic DNA of *M. roseus*. All growth experiments, as well as all qPCR measurements, were performed in triplicate. Transcription of the target oxidoreductase genes was normalized using the transcription level of both *rpoB* and *atpA*, which were transcribed at all the tested growth conditions in a similar ratio to each other (Supplementary Figure S1). Our final analysis was based on *rpoB*-normalized data.

## Results and Discussion

### Disclosure of the Aerobic Electron Transfer Chain Activity

Previous genome analysis revealed the presence of the major components of respiratory electron transfer chain (ETC) in *M. roseus* (Podosokorskaya et al., 2013): proton-translocating NADH-dehydrogenase complex I, membrane-bound succinate dehydrogenase/fumarate reductase, isoprenoid quinones, and quinol oxidizing alternative complex III (ACIII). To confirm the activity of this ETC in *M. roseus*, the influence of rotenone and HQNO on aerobic growth with maltose or acetate (a non-fermentable substrate) was evaluated. Each of the inhibitors completely arrested *M. roseus* growth at these conditions. Neither HQNO nor rotenone had affected fermentative growth of the organism on maltose without electron acceptors.

Rotenone is known as an inhibitor of type I NADH-dehydrogenases. This compound binds to the membranous quinone-binding subunit NuoH/Nqo8 or the interface of the subunits NuoB/D/H (Sazanov, 2012; Nichols, 2013).

2-*n*-heptyl-4-hydroxyquinoline-*N*-oxide can serve as an inhibitor of several quinone interacting enzymes. In particular, HQNO demonstrates strong inhibitory effect on complex II (Smirnova et al., 1995), as well as partial inhibition of ACIII (Refojo et al., 2010, 2012). Complete inhibition of *M. roseus* aerobic growth with HQNO suggests the blockage of the main electron flow leading to energy production. According to genomic analysis (Podosokorskaya et al., 2013) we can assume that the points of HQNO action could be the *M. roseus*complex II and the alternative complex III. Anyway, the observed inhibition of *M. roseus* aerobic growth with either rotenone or HQNO supports the presence and activity of at least two energy-transducing complexes – the complex I and, most probably, the alternative complex III.

**FIGURE 1:**
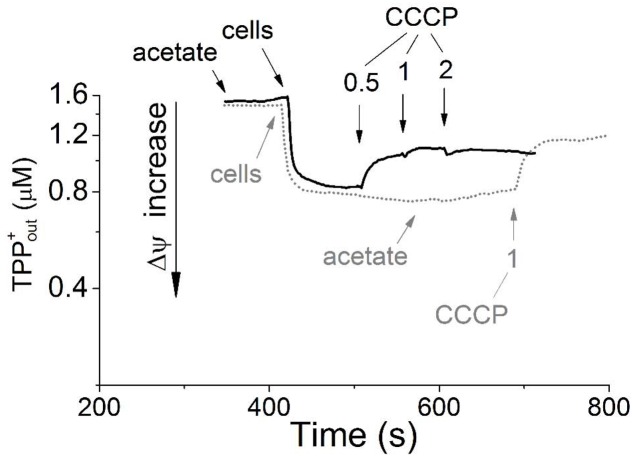
Generation of transmembrane electrical potential in aerobically grown resting cells of *Melioribacter roseus*. The incubation medium (pH 7.0) was the same as the growth medium with the exception that the growth factors were omitted, but TPP-Cl was added to the final concentration of 1.6 μM. Small arrows indicate time points of the following additions to the incubation mixture: the cells to achieve 3 mg ml^-1^ of total cell protein; sodium acetate to achieve 10 mM final concentration; and CCCP in micromolar concentrations as indicated in the panel. Membrane potential is shown in the intact cells preincubated without acetate before its addition (gray dotted line) and in the cells incubated for 5 min with acetate (black solid line). All measurements were carried out at 52°C.

The washed resting cells grown aerobically with acetate maintained a transmembrane electrical potential (Δψ) in the absence of added exogenous substrates, as was detected using the TPP^+^-selective electrode. The transmembrane potential dissipated upon the addition of 1 μM carbonyl cyanide *m*-chlorophenyl hydrazone (CCCP), which passively balances proton gradient across the cytoplasmic membrane (**Figure 1**, gray dotted line). Aliquots of these same cell preparations exhibited no respiration, as determined by a standard oxygen Clark-type electrode, unless they were supplied with exogenous energy substrates. In the presence of acetate, the cells began to respire with the oxygen consumption rate of 1.9 nmol O_2_ min^-1^ mg^-1^ of the cell protein (kinetic data are not presented). The Δψ value on the membranes of the respiring cells was maintained at the level observed in the absence of acetate (**Figure 1**, black solid line). Apparently, acetate serves as an external respiratory substrate in *M. roseus* cells, being presumably metabolized via the acetyl-CoA synthetase-catalyzed pathway and further through the TCA cycle predicted by genome analysis (Kadnikov et al., 2013). This pathway could directly fuel the respiratory ETC via the activity of succinate dehydrogenase, resulting in initiation of oxygen consumption by the cells. The presence of the respiratory complex II in *M. roseus* was previously revealed by genome analysis (Kadnikov et al., 2013) and its expression under aerobic condition is supported by our proteomic data (see below, Supplementary Table S2). In the presence of the respiratory substrate (acetate) and Δψ across the bacterial cell membranes (i.e., when the respiratory chain was in partially reduced state), cyanide inhibited respiratory activity of whole resting cells. The cyanide effect on oxygen respiration was comparatively high, as 50% inhibition was observed within 10 minutes in the presence of 35 μM KCN (without preincubation) with reductive substrate, and 65% inhibition was reached at 100 μM KCN within 20 min (Supplementary Figure S2). Noteworthily, our measurements were performed under optimal growth conditions of *M. roseus* (pH 7.0, 52°C), at which 99% cyanide (pK = 9.2) exists in the form of easily sublimating cyanic acid (boiling point 26°C). Consequently, the active concentration of cyanide in the incubation mixture for the respiratory activity measurements was to be well below the mentioned estimated value. Additionally, the inhibitory effect of cyanide on cytochrome oxidases depends on pH of the medium, membrane environment and RedOx state of the pentacoordinated oxygen-binding heme (that is heme *a*_3_/*o*_3_) in the enzyme reaction center (Wilson and Erecińska, 1978; Cooper and Brown, 2008). Considering these facts and reasons, and that our measurements were performed with the whole cells and incompletely oxidized heme proteins (when the cytochrome oxidases of clade A form unstable complexes with cyanide, i.e., reversibly bind it), we assume that the actual cyanide inhibitory effect on *M. roseus* cytochrome oxidases is much higher, than the apparent one registered in our study. Anyway, such an effect manifests the efficiency of cyanide as an inhibitor, thus suggesting that mainly the heme-copper oxidases comprise the terminus of the respiratory chain (Nichols, 2013) in the cells grown at atmospheric oxygen concentration.

Taken together, the results of inhibitory analysis of aerobic respiration and Δψ measurements performed with *M. roseus* whole cells clearly highlight the activity of aerobic respiratory electron transfer chain in the microorganism. The variety and functionality of three different oxygen reductases in *M. roseus* are analyzed in the next section on the basis of the genomic, proteomic and transcriptomic data.

### Identification of the Key Terminal Oxygen Reductases

**FIGURE 2:**
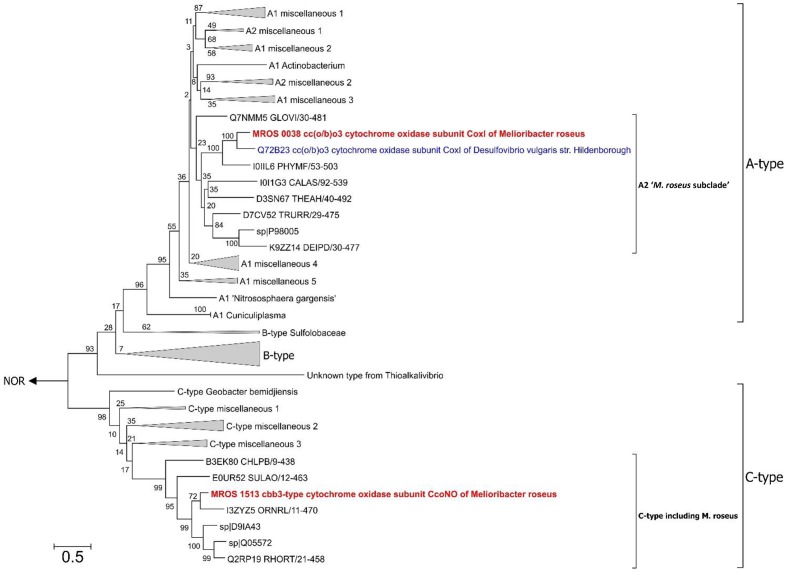
Maximum Likelihood phylogenetic tree of PF00115 domain (COX1 family, heme-copper oxygen reductases) sequences. Refer to the text for the description of A2 ‘*M. roseus* subclade’. After 50% sequence identity filtering of PF00115 sequences from the “seed” data-set (http://pfam.xfam.org/family/pf00115#tabview=tab3) a total of 112 sequences were used for the analysis. The tree with the highest log likelihood is shown. The bootstrap values (1000 replicates) are shown next to the branches. All positions with less than 95% site coverage were eliminated. There were a total of 367 positions in the final dataset. The tree was constructed in MEGA6 (Tamura et al., 2013). The “unknown group” was first introduced by Muntyan et al. (2015). Nitric oxide reductases (NOR) were used as an outgroup. CoxI protein of the *cc(o/b)o*_3_ cytochrome oxidase (A2-subtype) and CcoNO protein of the *cbb*_3_ oxygen reductase (C-type) of *M. roseus* are in bold red, and CoxI protein of oxygen detoxificating *cc(o/b)o*_3_ cytochrome oxidase of *D. vulgaris* str. Hildenborough is in bold blue. Bar is 0.5 substitutions per site.

During previous preliminary genome analysis of *M. roseus*, three putative terminal oxidoreductases were revealed: two heme-copper cytochrome *c* oxidases and a quinol oxidase of the *bd*-type (Podosokorskaya et al., 2013). Sequence analysis of the catalytic subunits MROS_0038 and MROS_1513 (CoxI and CcoNO, respectively) and the analysis of the genomic context of their genes (Podosokorskaya et al., 2013; Karnachuk et al., 2015) revealed that CoxI is highly similar (66% amino acid sequence identity at 97% query coverage) to the atypical heme-copper *cc(o/b)o_3_* cytochrome oxidase with an unusual heme content, which was recently described in a strict anaerobe *Desulfovibrio vulgaris* Hildenborough as a proton-translocating enzyme involved in oxygen detoxification (Ramel et al., 2013). The CcoNO of *M. roseus* was proposed to be a typical *cbb_3_*-type oxidoreductase with homologs in various Bacteroidetes, mainly aerobic ones (Podosokorskaya et al., 2013; Karnachuk et al., 2015). The phylogenetic analysis of *coxI* and *ccoNO* genes, performed within this work, generally reproduced the currently accepted phylogeny of heme-copper oxidoreductases (HCO; Sousa et al., 2012; Ducluzeau et al., 2014; Muntyan et al., 2015). The CoxI protein from *M. roseus* displayed close relation to the catalytic subunit of the *cc(o/b)o_3_* cytochrome oxidase described in *D. vulgaris*, while both enzymes appeared in the same distinct subclade of A-type oxidoreductases (**Figure 2**), which possess low affinity to O_2_ and are proposed to be best adapted to modern atmospheric oxygen concentration (Ducluzeau et al., 2014). Analysis of protein sequences from the ‘*M. roseus* subclade’ (**Figure 2**) with a web-based HCO classifying tool (Sousa et al., 2011, www.evocell.org/hco) recognized all of them as A2-subtype oxidoreductases possessing several peculiar residues in their proton channels (Sousa et al., 2012). The CcoNO protein of *M. roseus* fell within the clade of the authentic *cbb*_3_ oxidoreductases of C-type (**Figure 2**) notable for the mandatory Glu in the active center (Muntyan et al., 2015).

Sequence analysis of the catalytic subunit, CydA, of the *bd*-type quinol oxidase (MROS_0843) put this enzyme into the most widespread subfamily A of cytochromes *bd* with a “short Q-loop” between transmembrane helixes 6 and 7 (Borisov et al., 2011). Sequences from Bacteroidetes prevail in the first 100 best BLAST hits of MROS_0843 (UniProt database search on March 2017, Supplementary Table S3). In recent reviews, sporadic distribution of different cytochrome *bd*-type oxidases within the phylum Bacteroidetes was demonstrated and supposed to be a result of horizontal gene transfer. These cytochrome *bd* oxidases were proposed to perform various physiological functions apart from proton motive force generation, such as facilitating the colonization of O_2_-poor environments or detoxifying oxygen under oxidative stress and other stressful conditions (Borisov et al., 2011; Giuffrè et al., 2014).

**FIGURE 3:**
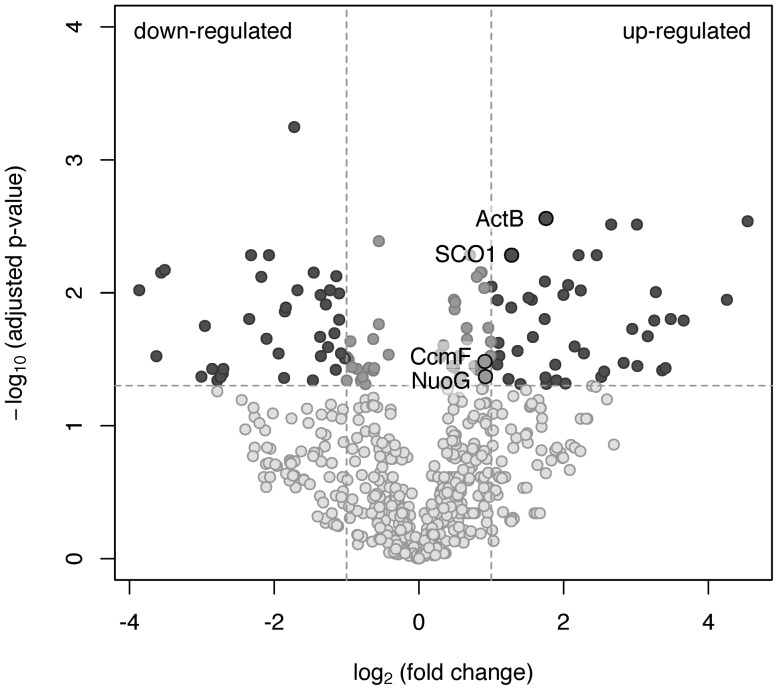
A volcano plot representation of the differentially expressed genes in a pairwise comparison of *M. roseus* cultures grown at aerobic and fermentative (strictly anaerobic) conditions. The significance cut-off was set to a *p*-value of 0.05 (-log_10_(adjusted *p*-value) >1.3), and the biological cut-off was set to a fold change of 2 (–1 ≥ log_2_(fold change) ≥1). The three colors differentiate genes with minor or insignificant changes in expression (grayish), statistically and biologically up-regulated or down-regulated genes (dark gray) and statistically but not biologically up-regulated or down-regulated genes (light gray). Proteins detected solely at aerobic or fermentative conditions are not the subjects of this analysis. Upregulated proteins captured in black circles are related to oxygen respiration (refer to the text for details).

To investigate which of the predicted oxidases and accessory proteins are involved in oxygen respiration at atmospheric O_2_ concentration, the results of a shotgun proteomic analysis of transmembrane and membrane-bound proteins were compared across two cultivation conditions: growth by aerobic respiration (at atmospheric O_2_ with acetate) and maltose fermentation under strictly anaerobic conditions (at negative *E_h_* of –130 mV vs SHE). Totally, at both growth conditions, each in three biological replicas, 1239 proteins were identified with a minimum of two unique peptides. The number of proteins identified per each sample is provided in Supplementary Tables S4–S6. For quantitative proteome analysis, exponentially modified protein abundance indexes (emPAIs) were calculated: 304 proteins were significantly different (adjusted *p*-value <0.05) between the cells grown by aerobic respiration and the cells grown by fermentation under strictly anaerobic conditions (Supplementary Table S2). Some proteins were identified reliably in aerobically grown cells only. Included among those proteins were (i) the catalytic subunit CoxI and the subunit CoxII of the *cc(o/b)o_3_* cytochrome oxidase, (ii) its redox partner class I soluble cytochrome *c_551_*/*c_552_* (MROS_0033), (iii) two subunits of the alternative complex III, ActC and fused ActDE, encoded in the same cluster with the *cc(o/b)o_3_* oxidase by MROS_0043 and MROS_0042, respectively, and (iv) the metal ion-binding subunit of copper-transporting ATPase, MROS_1511, proposed to participate in the biogenesis of heme-copper oxidases in *M. roseus* (Karnachuk et al., 2015). Upregulation at aerobic versus fermentative growth was statistically significant for the following proteins (**Figure 3**): (i) the catalytic subunit of the ACIII complex, ActB (encoded by MROS_0044), (ii) the NADH-dehydrogenase subunit, NuoG (encoded by MROS_2032), essential to provide the catalytic site for NADH oxidation in respiratory complex I, (iii) the SCO1/SenC domain protein encoded by MROS_0039, which is located in the same cluster (MROS_0034-0039) as the *cc(o/b)o_3_* oxidase genes and could determine the post-translational step in the accumulation of heme-copper oxidase subunits, CoxI and CoxII (Buchwald et al., 1991), (iv) the cytochrome *c* biogenesis protein F, encoded by MROS_0623 (CcmF). It should be mentioned that 3-fold upregulation at aerobic conditions was also significant in the case of subunit III of the *cbb_3_*-type oxygen reductase, while the catalytic subunit, CcoNO, of this complex was detected in only one biological replica, which does not allow for the prediction of the influence of aerobiosis on its abundance in the cells. Among proteins detected at fermentative conditions only (Supplementary Table S2) are hydrogenases of the [FeFe]-family (MROS_0634, 0635, 2480-2482, 2487, 2488), which were proposed to oxidize NADH to produce H_2_ during the fermentation of sugars (Kadnikov et al., 2013), and the subunit alpha of pyruvate:ferredoxin oxidoreductase (MROS_2663), which catalyzes the final step in the Embden-Meyerhof-Parnas pathway (Kletzin and Adams, 1996). This result seems logical considering that all of the proteins suppressed under aerobic conditions are involved in the fermentative catabolism of prokaryotes. Notably, insensitivity of the cytochrome *bd* oxidase to the change of growth conditions supports the prediction that this enzyme does not play a role of a terminal oxygen reductase during the aerobic growth of *M. roseus*.

Overall, the results of the proteomic analysis, in concordance with the abovementioned biochemical data, clearly indicate the involvement of complexes I, ACIII and the atypical heme-copper *cc(o/b)o_3_* cytochrome oxidase in aerobic respiration in *M. roseus*, what is supported by the expression pattern of the proteins involved in heme-copper enzymes’ biogenesis. To our knowledge, this is the first reported evidence at protein level of the involvement of ACIII in conjunction with the *cc(o/b)o_3_* cytochrome oxidase in aerobic respiration. The homolog of the *cc(o/b)o_3_* cytochrome oxidase of *M. roseus* has been comprehensively characterized only in *D. vulgaris*, in which the involvement of this enzyme in oxygen reduction coupled to proton motive force generation was demonstrated. However, no growth stimulation by oxygen was observed for this organism, and transcriptomic data only suggested a detoxifying role of this enzyme in the metabolism of the strict anaerobe, *D. vulgaris* (Lamrabet et al., 2011; Ramel et al., 2013). In general, our proteomic analysis revealed induction of the *cc(o/b)o_3_* cytochrome oxidase (MROS_0035-0038) during aerobic growth of *M. roseus* in comparison to fermentative growth, while the differences in expression of the *cbb_3_* cytochrome oxidase proteins (MROS_1513-1515) and the *bd*-type oxidase (MROS_0842-0843) were not reliably detected.

### Differentially Expressed Genes of Oxidoreductases at Various Growth Conditions

**FIGURE 4:**
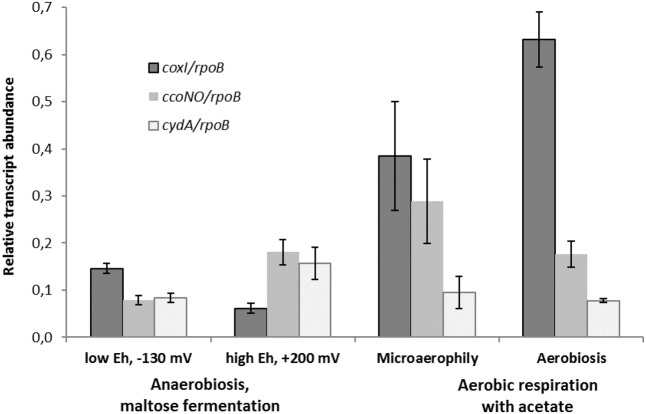
Relative transcript abundance of catalytic subunits of three different terminal oxygen reductases in aerobically or anaerobically (fermentatively) grown cells of *M. roseus*. Represented are the genes of catalytic subunits of the following terminal oxidases: *coxI* – heme-copper *cc(o/b)o*_3_ cytochrome oxidase, *ccoNO* – heme-copper *cbb*_3_-type oxidase and *cydA* – cytochrome *bd* oxidase. All the transcript abundances are normalized for the transcription level of the *rpoB* gene.

Comparative transcriptomics approach was used to confirm the findings of proteomic analysis and distinguish the metabolic roles of the two different HCOs and the cytochrome *bd* oxidase in *M. roseus.* Thus, the results of the RT-PCR analysis, targeted at *M. roseus* genes encoding the catalytic subunits of the three oxidoreductases (*coxI*, *ccoNO*, *cydA*), were compared across four different cultivation conditions: respiratory aerobic growth with acetate at atmospheric O_2_ concentration, microaerobic growth with acetate at 2% O_2_ in the gas phase, maltose fermentation at positive *E_h_* value of the medium (see Methods section) or at negative *E_h_* value (i.e., at strict anaerobiosis). An anaerobic medium with positive *E_h_* was used to assess possible involvement of the cytochrome oxidases in the oxidative stress response under the absence of oxygen in the gas phase but the presence of dissolved oxidized compounds. Strikingly, the genes of all three oxidases were transcribed at aerobic, microaerobic and both anaerobic growth conditions, although the normalized transcription level differed significantly (**Figure 4**). The highest normalized transcription level was observed for the *coxI* gene of the *cc(o/b)o_3_* cytochrome oxidase at aerobic conditions; the level decreased ca. 1.5-fold under microaerobic conditions (adjusted *p*-value 0.073, *p*-value 0.029) and significantly lowered 4 to 6-fold at anaerobic fermentative growth, both at negative and positive *E_h_* (adjusted *p*-value ≪ 0.05 for both pairwise comparisons). Different pattern was observed for *ccoNO* gene of the *cbb_3_*-type cytochrome oxidase, the transcription level of which was maximal at microaerobic cultivation conditions, did not statistically change at both aerobic or positive-*E_h_* anaerobic fermentative conditions and approached its minimum at strict anaerobiosis, at negative *E_h_* (adjusted *p*-value 0.028 for the comparison of maximal and minimal *ccoNO* transcription levels). Cytochrome *bd* oxidase demonstrated the lowest transcription levels among all three oxygen reductases at respiratory growth conditions (**Figure 4**). Although, transcript abundance of *cydA* gene was statistically indistinguishable at all tested growth conditions (adjusted *p*-values > 0.05 for all pairwise comparisons).

The results of transcriptomic studies demonstrate the key role of the A2-subtype *cc(o/b)o_3_* cytochrome oxidase as the terminal oxidoreductase in *M. roseus* aerobic respiration, while another heme-copper *cbb_3_*-type oxidase is supposed to play an auxiliary role in aerobic respiration, which, however, becomes important at microaerobic conditions when a higher affinity to O_2_ is needed to support the cell growth by oxygen respiration. Indeed, the oxidases of the *cbb_3_*-type possess a high affinity to oxygen (Pitcher et al., 2002) compared to the affinity measured for a *cc(o/b)o_3_* cytochrome oxidase in *D. vulgaris* (Ramel et al., 2013). Thus, the *cbb_3_* oxidase of *M. roseus* is likely to play a major role in aerobic respiration at microaerobic conditions or, alternatively, serve for oxygen scavenging upon anaerobic fermentative growth at positive *E_h_* (i.e., when dissolved O_2_ could be present in the environment at a concentration insufficient for the activity of the *cc(o/b)o_3_* cytochrome oxidase). Comparatively low transcription levels of the cytochrome *bd* oxidase at all tested conditions allows us to suggest that this enzyme mainly serves for the scavenging or detoxification of oxygen and is not directly involved in the aerobic energy metabolism of *M. roseus*. Of further note, the transcription of the *coxI* gene was significantly higher at strict anaerobiosis than at positive-*E_h_* fermentative conditions (adjusted *p*-value 0.028, **Figure 4**), what could reflect the similarity between the functional properties of the *cc(o/b)o_3_* cytochrome oxidase from *M. roseus* and its close homolog from the strict anaerobe, *D. vulgaris*, in which the detoxificating role of this oxidoreductase has been evidenced.

Interestingly, transcription of *atpA* (Supplementary Figure S1) and the genes of cytochrome oxidases (**Figure 4**) was still observed at strictly anaerobic conditions during maltose fermentation, although several enzymes involved in fermentative catabolism clearly were upregulated at these conditions, according to our proteomic analysis. The transcription of *atpA* during fermentation correlates with the expression data on four major F_0_F_1_-ATPase subunits (Supplementary Table S2) and could be explained in the view of probable functioning of the ATPase in the reverse direction of ATP hydrolysis for the dissipation of proton excess to prevent cytoplasm acidification. This function was proposed first for F_0_F_1_ ATPases in *Clostridium pasteurianum* and *Thermotoga maritima* pathways of acetogenic glucose fermentation, in which the key role was assigned to electron bifurcating [FeFe]-hydrogenases driving the thermodynamically unfavorable oxidation of NADH through the exergonic oxidation of ferredoxin to produce H_2_ (Buckel and Thauer, 2013). In the case of *M. roseus*, H_2_, acetate and CO_2_ are the main products of maltose fermentation (Podosokorskaya et al., 2013), and one of two putative [FeFe]-hydrogenases (MROS_0634) was upregulated during maltose fermentation. Accordingly, *M. roseus* is likely to possess a mode of fermentative catabolism similar to that proposed for *C. pasteurianum* and *T. maritima*, involving hydrolytic activity of F_0_F_1_ ATPase (Buckel and Thauer, 2013). Low-level transcription of cytochrome oxidases genes during fermentative growth could be explained by the “semper paratus” state of catabolic machinery in *M. roseus*, considering the instability of its natural environmental conditions (Podosokorskaya et al., 2013; Frank Y. et al., 2016), that is, the low biosynthesis level of key respiratory enzymes is probably sustained by a regulome of *M. roseus* in order to outcompete for electron acceptors upon sharp changes to the geochemical setting.

### Screening for Respiratory Arsenate Reductases

We have previously reported on arsenate respiration in growth experiments with *M. roseus* (Podosokorskaya et al., 2013). Preliminary genome analysis (Podosokorskaya et al., 2013) pointed out the genes of two oxidoreductase complexes belonging to the CISM superfamily (Duval et al., 2008). Phylogenetic analysis of their molybdopterin catalytic subunits MROS_1076 and MROS_1774 (Supplementary Figure S3), performed in the frames of the current work, revealed their affiliation to tetrathionate- (Ttr) and polysulfide/thiosulfate- (Psr/Phs) reductase branches, respectively, but not to dissimilatory arsenate reductases (Arr). According to our (Supplementary Figure S3) and previous (Duval et al., 2008; Sorokin et al., 2016) reconstructions, the Ttr clade originates from the same root as the Arr clade, which, in its turn, has a common ancestor with the Psr/Phs cluster. To the moment, the only organism reported to use a non-Arr-type oxidoreductase to respire arsenate is *Pyrobaculum aerophilum*. This hyperthermophilic archaeon, similarly to *M. roseus*, possesses Ttr- and Psr/Phs-type, but not Arr-type, oxidoreductases of the CISM superfamily (Cozen et al., 2009).

Considering that no other candidates for arsenate respiration were found in *M. roseus*, the transcription pattern of molybdopterin catalytic subunits MROS_1076 and MROS_1774 was studied for the cells grown by respiration with acetate as the electron donor and arsenate as the sole electron acceptor under anaerobic cultivation conditions. The cells, grown with acetate under aerobic or microaerobic conditions and those grown by maltose fermentation under strict anaerobiosis (*E_h_* –130 mV), were used as negative controls. Two sets of primers for *M. roseus* genes, encoding the molybdopterin catalytic subunits, were designed, and normalized transcription levels were compared across all four cultivation conditions (arsenate, aerobic, microaerobic respiration, and maltose fermentation).

**FIGURE 5:**
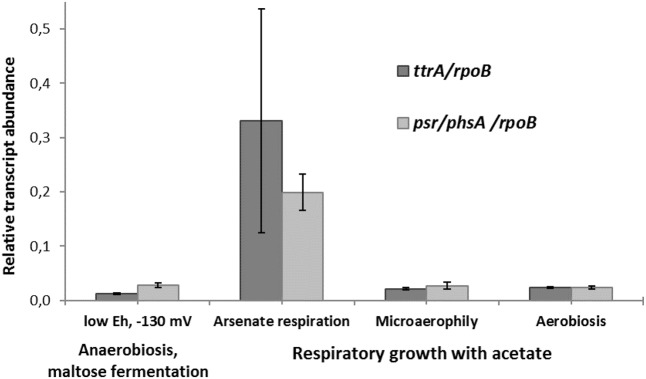
Relative transcript abundance of *ttrA* and *psr*/*phsA* genes involved in arsenate respiration of *M. roseus* grown at different growth conditions. Represented are the genes of catalytic subunits of the following CISM family oxidoreductases: *ttrA* – tetrathionate-like reductase, presumably involved in arsenate detoxification cascade, and *psr*/*phsA* – polysulfide/thiosulfate-like reductase, presumed to serve as the terminal dissimilatory arsenate reductase. All the transcript abundances are normalized for the transcription level of the *rpoB* gene.

**FIGURE 6:**
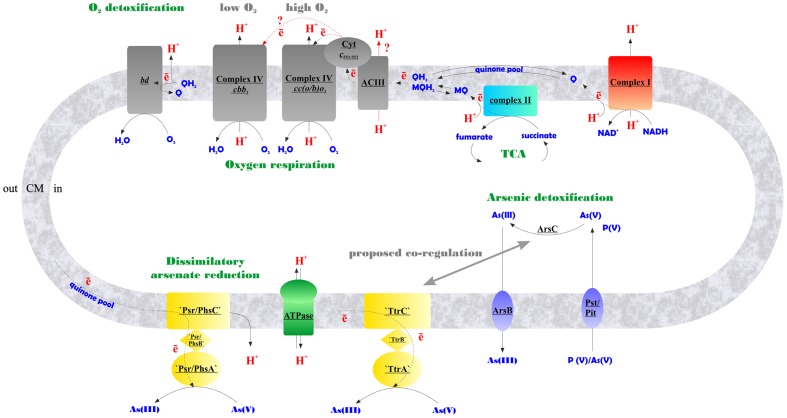
Schematic representation of aerobic and arsenate respiratory pathways of *M. roseus* summarized from our data. Contracted notations: bd – quinol oxidase of the *bd*-type; ACIII – alternative respiratory complex III; Cyt *c*_551/552_ – soluble monoheme *c*-type cytochrome transferring electrons from ACIII to heme-copper terminal oxidoreductases of A-type – *cc(o/b)o*_3_, or C-type – *cbb*_3_, upregulated at atmospheric O_2_ concentration or at microaerobic conditions, respectively; ‘Psr/PhsABC,’ polysulfide/thiosulfate-like reductase complex of CISM family; ‘TtrABC’, tetrathionate-like reductase complex of CISM family (A, B and C stand for molybdopterin-containing catalytic subunits, Fe–S subunits, and membrane-bound subunits of CISM complexes, respectively); ArsC, detoxifying arsenate reductase; ArsB, arsenite efflux pump; Pst/Pit, phosphate transporter systems, non-specifically transporting arsenate into the cell (Paez-Espino et al., 2009), encoded by MROS_2637-2640, and MROS_1319 in *M. roseus*; TCA, tricarboxylic acid cycle; Q/MQ, oxidized quinones (in particular, menaquinones); QH_2_/MQH_2_, reduced quinones/menaquinones; CM, cytoplasmic membrane. Firm lines with arrows indicate proton flows, dotted lines indicate electron flows, red lines with red query marks indicate putative electron and proton transfer pathways.

The transcription of *ttrA* and *psr/phsA* genes was almost negligible in the absence of arsenate but dramatically increased in cells grown with arsenate (**Figure 5**). These results suggest that both ‘Psr/Phs’ and ‘Ttr’ molybdopterin oxidoreductases are involved in arsenate respiration. Yet we should note, that only the induction of *psrA/phsA* was clearly statistically distinguishable (adjusted *p*-value 0.03) in arsenate-grown cells of *M. roseus*. Interestingly, despite the presence of Ttr- and Psr/Phs-type enzymes as candidate arsenate reductases in both *P. aerofilum* and *M. roseus* (Supplementary Figure S3), As(V) did induce only one molybdopterin oxidoreductase in *P. aerofilum* – the TtrA (PAE1265, Cozen et al., 2009), while in *M. roseus* both the ‘Ttr’ and the ‘Psr/Phs’ oxidoreductases were induced with arsenate and the ‘Psr/Phs’ induction was more pronounced (**Figure 5**). Although, we cannot exclude participation of the ‘Ttr’ oxidoreductase in arsenate metabolism of *M. roseus*, considering occurrence of transcriptional response of the *ttrA* gene to arsenate and its peculiar genomic neighborhood. Encoded right upstream of the *ttr* locus in *M. roseus* genome is an ‘ArrTSR’ two-component regulatory system (Saltikov, 2011), including a phosphorylated response regulator (‘ArrR’, MROS_1070), a sensory histidine kinase (‘ArrS’, MROS_1069) and a hypothetical protein (MROS_RS05510 according to the recent NCBI RefSeq reannotation), homologous to a periplasmic phosphonate binding protein ‘ArrT’ (Saltikov, 2011), a part of phosphonate ABC transporters (Alicea et al., 2011). The ‘ArrTSR’ system is supposed to induce transcription of dissimilatory arsenate reductases in response to periplasmic arsenate via the ‘ArrR’ regulator (Saltikov, 2011). Finally, in *M. roseus* in close vicinity to *arrT* gene is an ars-type arsenic resistance locus *arsRPCB* (MROS_1063-1067, Kadnikov et al., 2013), encoding detoxificating glutathione:arsenate oxidoreductase ArsC, an arsenite exporter ArsB and a specific regulator ArsR, which induces transcription of *arsPCB* in the presence of arsenite (Saltikov, 2011). In contrast to *ttr*, the *psr*/*phs* locus is preceded by the gene of MerR-like transcription activator (MROS_1773), which can respond to heavy metal ions and chemical stresses (Brown et al., 2003). Peculiarities of genomic environment of *ttrA* and *psrA*/*phsA* genes allow us to hypothesize that ‘PsrA/PhsA’ and ‘TtrA’ enzymes play different roles in arsenate respiration of *M. roseus*. We propose the ‘PsrA/PhsA’ to be the major arsenate reductase, as its transcription could be directly induced by As(V) and such an induction is supported by our experimental data. On the other hand, ‘TtrA’ could act as an auxiliary arsenate reductase which activity could be co-regulated with arsenic detoxification complex ArsPCB. Thus, our results indicate utilization of two non-Arr arsenate reductases by *M. roseus* in arsenic respiratory metabolism (**Figure 6**), yet further studies are definitely needed to cast light on their exact physiological functions.

## Conclusion

In this study, we present multiple evidences on the involvement of atypical respiratory enzymes in the energy metabolism of an extremophilic bacterium representing deep phylogenetic lineage and originating from one of Eurasian deep subsurface environments, which remained separated from the rest of the biosphere for several geological periods.

By using a combination of biochemical, bioinformatic, transcriptomic and proteomic approaches, we have evidenced the functioning of the electron transfer chain during aerobic respiration of *M. roseus*, which is comprised of NADH-dehydrogenase I, respiratory complex II, alternative complex III and an atypical A2-subtype *cc(b/o)o_3_* cytochrome oxidase. The *cc(b/o)o_3_* cytochrome oxidase has been previously characterized exclusively in *D. vulgaris*, where it was shown to serve only for oxygen detoxification. Here is the first evidence of the involvement of this enzyme in aerobic respiration. Furthermore, a wide distribution of homologs of this type of enzyme among aerobes (Lamrabet et al., 2011) might suggest its involvement in oxygen respiration in many of these microorganisms. Based on our results, we propose the metabolic scheme of aerobic respiration in *M. roseus* (**Figure 6**), in which the *cc(b/o)o_3_* cytochrome oxidase is the major terminal oxidoreductase upon atmospheric O_2_ concentration, while at microaerobic growth conditions another heme-copper oxidase, belonging to the *cbb_3_*-type, solos in energy transduction in this organism. The third oxidoreductase detected in *M. roseus*—the cytochrome *bd* oxidase—most likely serves for oxygen scavenging at both aerobic and anaerobic growth conditions. Our transcriptomic studies have supported comparative genomics predictions on participation of two molybdopterin oxidoreductases, not belonging to the Arr clade, in arsenate respiration. We further hypothesize that these two molybdoenzymes are involved differently in arsenic metabolism of *M. roseus*: while one of them acts as the major dissimilatory arsenate reductase, the other is likely to be linked with arsenic detoxification pathway (**Figure 6**).

## Author Contributions

SG, IK, EB-O convened the research. SG, IK, AM, DA, VG designed the research. SG, IK, OP, AM, MK, MM, IA, IB performed experimental work. SG, IK, MM, OP, IA, EB-O wrote the manuscript.

## Conflict of Interest Statement

The authors declare that the research was conducted in the absence of any commercial or financial relationships that could be construed as a potential conflict of interest.

## Footnotes

1 http://www.cbs.dtu.dk/services

2 http://www.psort.org/psortb/

3 http://phobius.sbc.su.se/

## References

[B1] Abascal F. Zardoya R. Posada D. (2005). ProtTest: selection of best-fit models of protein evolution. *Bioinformatics* 21 2104–2105. 10.1093/bioinformatics/bti263 15647292

[B2] Alicea I. Marvin J. S. Miklos A. E. Ellington A. D. Looger L. L. Schreiter E. R. (2011). Structure of the *Escherichia coli* phosphonate binding protein PhnD and rationally optimized phosphonate biosensors. *J. Mol. Biol.* 414 356–369. 10.1016/j.jmb.2011.09.047 22019591 PMC5320564

[B3] Benjamini Y. Hochberg Y. (1995). Controlling the false discovery rate: a practical and powerful approach to multiple testing. *J. R. Stat. Soc. Series B Stat. Methodol.* 57 289–300.

[B4] Borisov V. B. Gennis R. B. Hemp J. Verkhovsky M. I. (2011). The cytochrome *bd* respiratory oxygen reductases. *Biochim. Biophys. Acta* 1807 1398–1413. 10.1016/j.bbabio.2011.06.016 21756872 PMC3171616

[B5] Brown N. L. Stoyanov J. V. Kidd S. P. Hobman J. L. (2003). The MerR family of transcriptional regulators. *FEMS Microbiol. Rev.* 27 145–163. 10.1016/S0168-6445(03)00051-2 12829265

[B6] Buchwald P. Krummeck G. Rodel G. (1991). Immunological identification of yeast SCO1 protein as a component of the inner mitochondrial membrane. *Mol. Gen. Genet.* 229 413–420. 10.1007/BF00267464 1944230

[B7] Buckel W. Thauer R. K. (2013). Energy conservation via electron bifurcating ferredoxin reduction and proton/Na+ translocating ferredoxin oxidation. *Biochim. Biophys. Acta* 1827 94–113. 10.1016/j.bbabio.2012.07.002 22800682

[B8] Cooper C. E. Brown G. C. (2008). The inhibition of mitochondrial cytochrome oxidase by the gases carbon monoxide, nitric oxide, hydrogen cyanide and hydrogen sulfide: chemical mechanism and physiological significance. *J. Bioenerg. Biomembr.* 40 533–539. 10.1007/s10863-008-9166-6 18839291

[B9] Cozen A. E. Weirauch M. T. Pollard K. S. Bernick D. L. Stuart J. M. Lowe T. M. (2009). Transcriptional map of respiratory versatility in the hyperthermophilic Crenarchaeon *Pyrobaculum aerophilum*. *J. Bacteriol.* 191 782–794. 10.1128/JB.00965-08 19047344 PMC2632070

[B10] Ducluzeau A.-L. Schoepp-Cothenet B. van Lis R. Baymann F. Russell M. J. Nitschke W. (2014). The evolution of respiratory O2/NO reductases: an out-of-the-phylogenetic-box perspective. *J. R. Soc. Interface* 11:20140196 10.1098/rsif.2014.0196 PMC4233682 24968694

[B11] Duval S. Ducluzeau A.-L. Nitschke W. Schoepp-Cothenet B. (2008). Enzyme phylogenies as markers for the oxidation state of the environment: the case of respiratory arsenate reductase and related enzymes. *BMC Evol. Biol.* 8:206 10.1186/1471-2148-8206 PMC2500031 18631373

[B12] Elshahed M. S. Senko J. M. Najar F. Z. Kenton S. M. Roe B. A. Dewers T. A. (2003). Bacterial diversity and sulfur cycling in a mesophilic sulfide-rich spring. *Appl. Environ. Microbiol.* 69 5609–5621. 10.1128/AEM.69.9.5609-5621.2003 12957951 PMC194924

[B13] Frank Y. Banks D. Avakian M. Antsiferov D. Kadychagov P. Karnachuk O. (2016). Firmicutes is an important component in water-injected and pristine oil reservoirs; Western Siberia Russia. *Geomicrobiol. J.* 33 387–400. 10.1080/01490451.2015.1045635

[B14] Frank Y. A. Kadnikov V. V. Gavrilov S. N. Banks D. Gerasimchuk A. L. Podosokorskaya O. A. (2016). Stable and variable parts of microbial community in siberian deep subsurface thermal aquifer system revealed in a long-term monitoring study. *Front. Microbiol.* 7:2101 10.3389/fmicb.2016.02101 PMC5187383 28082967

[B15] Gennis R. B. (2013). “Bacterial respiratory oxygen reductases,” in *Encyclopedia of Biophysics*, ed. Roberts G. C. K. (Berlin: Springer-Verlag), 178–181. 10.1007/978-3-642-16712-6_33

[B16] Giuffrè A. Borisov V. B. Arese M. Sarti P. Forte E. (2014). Cytochrome *bd* oxidase and bacterial tolerance to oxidative and nitrosative stress. *Biochim. Biophys. Acta* 1837 1178–1187. 10.1016/j.bbabio.2014.01.016 24486503

[B17] Huang Y. Niu B. Gao Y. Fu L. Li W. (2010). CD-HIT Suite: a web server for clustering and comparing biological sequences. *Bioinformatics* 26 680–682. 10.1093/bioinformatics/btq003 20053844 PMC2828112

[B18] Iino T. Mori K. Uchino Y. Nakagawa T. Harayama S. Suzuki K. (2010). *Ignavibacterium album* gen. nov., sp. nov., a moderately thermophilic anaerobic bacterium isolated from microbial mats at a terrestrial hot spring and proposal of *Ignavibacteria classis* nov., for a novel lineage at the periphery of green sulfur bacteria. *Int. J. Syst. Evol. Microbiol.* 60 1376–1382. 10.1099/ijs.0.012484-0 19671715

[B19] Ishihama Y. Oda Y. Tabata T. Sato T. Nagasu T. Rappsilber J. (2005). Exponentially modified protein abundance index (emPAI) for estimation of absolute protein amount in proteomics by the number of sequenced peptides per protein. *Mol. Cell. Proteomics* 4 1265–1272. 10.1074/mcp.M500061-MCP200 15958392

[B20] Kadnikov V. V. Mardanov A. V. Podosokorskaya O. A. Gavrilov S. N. Kublanov I. V. Beletsky A. V. (2013). Genomic analysis of *Melioribacter roseus*, facultatively anaerobic organotrophic bacterium representing a novel deep lineage within Bacteriodetes/Chlorobi group. *PLoS ONE* 8:e53047 10.1371/journal.pone.0053047 PMC3534657 23301019

[B21] Kamo N. Muratsugu M. Hongoh R. Kobatake Y. (1979). Membrane potential of mitochondria measured with an electrode sensitive to tetraphenyl phosphonium and relationship between proton electrochemical potential and phosphorylation potential in steady state. *J. Membr. Biol.* 49 105–121. 10.1007/BF01868720 490631

[B22] Karnachuk O. V. Gavrilov S. N. Avakyan M. R. Podosokorskaya O. A. Frank Y. A. Bonch-Osmolovskaya E. A. (2015). Diversity of copper proteins and copper homeostasis systems in *Melioribacter roseus*, a facultatively anaerobic thermophilic member of the new phylum Ignavibacteriae. *Microbiology* 84 135–143. 10.1134/S0026261715020058 26263622

[B23] Kato S. Ikehata K. Shibuya T. Urabe T. Ohkuma M. Yamagishi A. (2015). Potential for biogeochemical cycling of sulfur, iron and carbon within massive sulfide deposits below the seafloor. *Environ. Microbiol.* 17 1817–1835. 10.1111/1462-2920.12648 25330135

[B24] Katoh K. Misawa K. Kuma K. Miyata T. (2002). MAFFT: a novel method for rapid multiple sequence alignment based on fast Fourier transform. *Nucleic Acids Res.* 30 3059–3066. 10.1093/nar/gkf436 12136088 PMC135756

[B25] Kletzin A. Adams M. W. (1996). Molecular and phylogenetic characterization of pyruvate and 2-ketoisovalerate ferredoxin oxidoreductases from *Pyrococcus furiosus* and pyruvate ferredoxin oxidoreductase from *Thermotoga maritima*. *J. Bacteriol.* 178 248–257. 10.1128/jb.178.1.248-257.1996 8550425 PMC177646

[B26] Lamrabet O. Pieulle L. Aubert C. Mouhamar F. Stocker P. Dolla A. (2011). Oxygen reduction in the strict anaerobe *Desulfovibrio vulgaris* Hildenborough: characterization of two membrane-bound oxygen reductases. *Microbiology* 157 2720–2732. 10.1099/mic.0.049171-0 21737501

[B27] Mattar S. Engelhard M. (1997). Cytochrome ba3 from *Natronobacterium pharaonis*. *Eur. J. Biochem.* 250 332–341. 10.1111/j.1432-1033.1997.0332a.x 9428682

[B28] Muntyan M. S. Cherepanov D. A. Malinen A. M. Bloch D. A. Sorokin D. Y. Severina I. I. (2015). Cytochrome cbb3 of *Thioalkalivibrio* is a Na+-pumping cytochrome oxidase. *Proc. Natl. Acad. Sci. U.S.A.* 112 7695–7700. 10.1073/pnas.1417071112 26056262 PMC4485098

[B29] Muntyan M. S. Morozov D. A. Klishin S. S. Khitrin N. V. Kolomijtseva G. Y. (2012). Evaluation of the electrical potential on the membrane of the extremely alkaliphilic bacterium *Thioalkalivibrio*. *Biochemistry (Moscow)* 77 917–924. 10.1134/S0006297912080135 22860914

[B30] Nichols D. G. (2013). *Bioenergetics*, 4th Edn. Amsterdam: Academic Press.

[B31] Paez-Espino J. T. Tamames J. de Lorenzo V. Canovas D. (2009). Microbial responses to environmental arsenic. *Biometals* 22 117–130. 10.1007/s10534-008-9195-y 19130261

[B32] Pitcher R. S. Brittain T. Watmough N. J. (2002). Cytochrome cbb(3) oxidase and bacterial microaerobic metabolism. *Biochem. Soc. Trans.* 30 653–658. 10.1042/bst0300653 12196157

[B33] Podosokorskaya O. A. Kadnikov V. V. Gavrilov S. N. Mardanov A. V. Merkel A. Y. Karnachuk O. V. (2013). Characterization of *Melioribacter roseus* gen. nov., sp. nov., a novel facultatively anaerobic thermophilic cellulolytic bacterium from the class Ignavibacteria, and a proposal of a novel bacterial phylum Ignavibacteriae. *Environ. Microbiol.* 15 1759–1771. 10.1111/1462-2920.12067 23297868

[B34] Ramel F. Amrani A. Pieulle L. Lamrabet O. Voordouw G. Seddiki N. (2013). Membrane-bound oxygen reductases of the anaerobic sulfate-reducing *Desulfovibrio vulgaris* Hildenborough: roles in oxygen defense and electron link with periplasmic hydrogen oxidation. *Microbiology* 159 2663–2673. 10.1099/mic.0.071282-0 24085836

[B35] Rauhamäki V. Bloch D. A. Wikström M. (2012). Mechanistic stoichiometry of proton translocation by cytochrome *cbb*_3_. *Proc. Natl. Acad. Sci. U.S.A.* 109 7286–7291. 10.1073/pnas.1202151109 22529361 PMC3358842

[B36] Rauhamäki V. Wikström M. (2014). The causes of reduced proton-pumping efficiency in type B and C respiratory heme-copper oxidases, and in some mutated variants of type A. *Biochim. Biophys. Acta* 1837 999–1003. 10.1016/j.bbabio.2014.02.020 24583065

[B37] Refojo P. N. Teixeira M. Pereira M. M. (2010). The alternative complex III of *Rhodothermus marinus* and its structural and functional association with caa3 oxygen reductase. *Biochim. Biophys. Acta* 1797 1477–1482. 10.1016/j.bbabio.2010.02.029 20206595

[B38] Refojo P. N. Teixeira M. Pereira M. M. (2012). The alternative complex III: properties and possible mechanisms for electron transfer and energy conservation. *Biochim. Biophys. Acta* 1817 1852–1859. 10.1016/j.bbabio.2012.05.003 22609325

[B39] Rothery R. A. Workun G. J. Weiner J. H. (2008). The prokaryotic complex iron–sulfur molybdoenzyme family. *Biochim. Biophys. Acta* 1778 1897–1929. 10.1016/j.bbamem.2007.09.002 17964535

[B40] Saltikov C. W. (2011). “Regulation of arsenic metabolic pathways in prokaryotes,” in *Microbial Metal and Metalloid Metabolism: Advances and Applications*, eds Stolz J. F. Oremland R. S. (Washington, DC: ASM Press), 195–210. 10.1128/9781555817190.ch11

[B41] Sazanov L. A. (2012). “Structural perspective on respiratory complex I,” in *Structure and Function of NADH: Ubiquinone Oxidoreductase*, ed. Sazanov L. (Dordrecht: Springer), 10.1007/978-94-007-4138-6

[B42] Smirnova I. A. Hägerhäll C. Konstantinov A. A. Hederstedt L. (1995). HOQNO interaction with cytochrome b in succinate: menaquinone oxidoreductase from *Bacillus subtilis*. *FEBS Lett.* 359 23–26. 10.1016/0014-5793(94)01442-4 7851524

[B43] Sorokin D. Y. Kublanov I. V. Gavrilov S. N. Rojo D. Roman R. Golyshin P. N. (2016). Elemental sulfur and acetate can support life of a novel strictly anaerobic haloarchaeon. *ISME J.* 10 240–252. 10.1038/ismej.2015.79 25978546 PMC4681856

[B44] Sousa F. L. Alves R. J. Pereira-Leal J. B. Teixeira M. Pereira M. M. (2011). A bioinformatics classifier and database for heme–copper oxygen reductases. *PLoS ONE* 6:e19117 10.1371/journal.pone.0019117 PMC3084760 21559461

[B45] Sousa F. L. Alves R. J. Ribeiro M. A. Pereira-Leal J. B. Teixeira M. Pereira M. M. (2012). The superfamily of heme-copper oxygen reductases: types and evolutionary considerations. *Biochim. Biophys. Acta* 1817 629–637. 10.1016/j.bbabio.2011.09.020 22001780

[B46] Tamura K. Stecher G. Peterson D. Filipski A. Kumar S. (2013). MEGA6: molecular evolutionary genetics analysis version 6.0. *Mol. Biol. Evol.* 30 2725–2729. 10.1093/molbev/mst197 24132122 PMC3840312

[B47] Tiodjio R. E. Sakatoku A. Nakamura A. Tanaka D. Fantong W. Y. Tchakam K. B. (2014). Bacterial and archaeal communities in Lake Nyos (Cameroon, Central Africa). *Sci. Rep.* 4:6151 10.1038/srep06151 PMC4139950 25141868

[B48] Vizcaíno J. A. Deutsch E. W. Wang R. Csordas A. Reisinger F. Ríos D. (2014). ProteomeXchange provides globally coordinated proteomics data submission and dissemination. *Nat. Biotechnol.* 32 223–226. 10.1038/nbt.2839 24727771 PMC3986813

[B49] Wikström M. K. (1977). Proton pump coupled to cytochrome c oxidase in mitochondria. *Nature* 266 271–273. 10.1038/266271a0 15223

[B50] Wilson D. F. Erecińska M. (1978). Ligands of cytochrome c oxidase. *Methods Enzymol.* 53 191–201. 10.1016/S0076-6879(78)53024 213678

[B51] Ziganshin R. H. Ivanova O. M. Lomakin Y. A. Belogurov A. A. Jr. Kovalchuk S. I. Azarkin I. V. (2016). The pathogenesis of the demyelinating form of Guillain-Barre syndrome: proteopeptidomic and immunological profiling of physiological fluids. *Mol. Cell. Proteomics* 15 2366–2378. 10.1074/mcp.M115.056036 27143409 PMC4937510

