# Supplementary material for: Respiratory Pathways Reconstructed by Multi-Omics Analysis in Melioribacter roseus, Residing in a Deep Thermal Aquifer of the West-Siberian Megabasin
Source: Front Microbiol. 2017 Jun 30;8:1228. doi: 10.3389/fmicb.2017.01228 (PMC5492636; doi:10.3389/fmicb.2017.01228)
Supplement: Supplementary file 4 [file Data_Sheet_1.docx]

**Respiratory pathways reconstructed by multi-omics analysis in *Melioribacter roseus*, residing in a deep thermal aquifer of the West-Siberian megabasin**

**Sergey Gavrilov, Olga Podosokorskaya, Dmitry Alexeev, Alexander Merkel, Maria Khomyakova, Maria Muntyan, Ilya Altukhov, Ivan Butenko, Elizaveta Bonch-Osmolovskaya, Vadim Govorun and Ilya Kublanov**

Running title: **Respiratory pathways in *Melioribacter roseus***

**Supplementary material**

(except Tables S2, S5 and S6)

**Table S1.** RT-PCR primers designed and used in the study.

| **Primer name** | **Primer sequence (5′–3′)** | **Target gene** | **Product length, bp** | **Annealing temp., °C** |
| --- | --- | --- | --- | --- |
| MR_atpA_F | CGGAACGGTGTTGCAGGTAG | *atpA* | 152 | 65 |
| MR_atpA_R | ACTCGCCAAAGAGAACGCAG |  |  |  |
| MR_rpoB_F | CATCGACCATCTGGGCAACA | *rpoB* | 166 | 65 |
| MR_rpoB_R | GCTGACCGTTCTGGCATTGA |  |  |  |
| MR_coxI_F | ATCGCAATTCCCACGCTAATC | *coxI* | 179 | 65 |
| MR_coxI_R | TCGTTCACAGGCACATAGAGAG |  |  |  |
| MR_ccoNO_F | GGCTGGAACGGACTTCTGAC | *ccoNO* | 150 | 65 |
| MR_ccoNO_R | CGACCAATACATCGGCACCA |  |  |  |
| MR_cydA_F | GGCGGAAGTGGGAAGACAA | *cydA* | 145 | 65 |
| MR_cydA_R | AAGGAACAACGAACCGAGCA |  |  |  |
| MR_ttrA_F | TCCATTTGAGCAAGCCATTCAG | *ttrA* | 124 | 65 |
| MR_ttrA_R | AACATCGGCATCTCGCAAAG |  |  |  |
| MR_phsA_F | GCCTTCGTTTGCTCAATGCC | *psr/phsA* | 145 | 65 |
| MR_phsA_R | GTGCATATTCTCGCCCAGGT |  |  |  |

**Table S2** (presented as a separate MS Excel file).

Label-free protein quantification.

**Table S3.** Top 100 best BLAST hits of *M. roseus* CydA protein by UniProt database search

on March 2017 with 0,0001 E-value threshold and filtering for low complexity. Sequences lacking phylum-level ascription were excluded.

| # | Entry | E-value | Identity | Protein existence | Phylum | Species |
| --- | --- | --- | --- | --- | --- | --- |
| 1 | A0A1J4U5P2 | 0.0 | 75.3% | Predicted | Ignavibacteriae | Ignavibacteria bacterium CG1_02_37_35 |
| 2 | A0A0S8H7D9 | 0.0 | 62.6% | Predicted | Gemmatimonadetes | Gemmatimonas sp. SM23_52 |
| 3 | A0A1F3C2R7 | 0.0 | 62.8% | Predicted | Proteobacteria | Anaeromyxobacter sp. RBG_16_69_14 |
| 4 | B8J7L4 | 0.0 | 63.4% | Predicted | Proteobacteria | Anaeromyxobacter dehalogenans |
| 5 | B4UE40 | 0.0 | 63.4% | Predicted | Proteobacteria | Anaeromyxobacter sp. (strain K) |
| 6 | Q2IFY6 | 0.0 | 62.7% | Predicted | Proteobacteria | Anaeromyxobacter dehalogenans |
| 7 | A0A0D6QQP9 | 0.0 | 62.2% | Predicted | Proteobacteria | Anaeromyxobacter sp. PSR-1 |
| 8 | A0A0S8GNZ3 | 0.0 | 60.3% | Predicted | Planctomycetes | Phycisphaerae bacterium SM23_30 |
| 9 | A0A1F9CC48 | 0.0 | 58.8% | Predicted | Proteobacteria | Deltaproteobacteria bacterium RBG_16_49_23 |
| 10 | A0A1G3Y4R9 | 0.0 | 57.8% | Predicted | Nitrospirae | Thermodesulfovibrio sp. RBG_19FT_COMBO_42_12 |
| 11 | A0A1G1HUY3 | 3.1e-177 | 56.3% | Predicted | Nitrospirae | Nitrospirae bacterium RIFCSPLOW2_12_42_9 |
| 12 | W0JK46 | 6.3e-164 | 55.2% | Predicted | Proteobacteria | Desulfurella acetivorans |
| 13 | B0TEW6 | 5e-162 | 54.9% | Predicted | Firmicutes | Heliobacterium modesticaldum |
| 14 | A0A1F3VNN8 | 6.9e-161 | 54.3% | Predicted | Proteobacteria | Bdellovibrionales bacterium RIFOXYC1_FULL_37_79 |
| 15 | A0A0M2U280 | 3.4e-159 | 53.3% | Predicted | Firmicutes | Clostridiales bacterium PH28_bin88 |
| 16 | A0A0L6W1X9 | 3.1e-153 | 51.7% | Predicted | Firmicutes | Thermincola ferriacetica |
| 17 | D5XD71 | 8.7e-153 | 51.5% | Predicted | Firmicutes | Thermincola potens |
| 18 | A0A1G0QQH3 | 5.5e-152 | 51.5% | Predicted | Ignavibacteriae | Ignavibacteria bacterium GWC2_36_12 |
| 19 | A0A1G0NUR7 | 5.5e-152 | 51.5% | Predicted | Ignavibacteriae | Ignavibacteria bacterium GWA2_35_9 |
| 20 | Q3A9E5 | 1.2e-150 | 50.6% | Predicted | Firmicutes | Carboxydothermus hydrogenoformans |
| 21 | I0AGM5 | 3.1e-150 | 51.0% | Predicted | Ignavibacteriae | Ignavibacterium album |
| 22 | A0A1G0VE20 | 2e-149 | 49.4% | Predicted | Ignavibacteriae | Ignavibacteria bacterium RIFOXYB2_FULL_37_11 |
| 23 | A0A1G1BAA4 | 2e-149 | 49.4% | Predicted | Ignavibacteriae | Melioribacter sp. GWF2_38_21 |
| 24 | A0A1G0SUH6 | 4.4e-149 | 50.8% | Predicted | Ignavibacteriae | Ignavibacteria bacterium RBG_16_34_14 |
| 25 | A0A1G1E2A1 | 2.2e-150 | 59.4% | Predicted | Nitrospirae | Nitrospirae bacterium GWA2_42_11 |
| 26 | A0A0P7XQ43 | 2.9e-148 | 52.2% | Predicted | Bacteroidetes | Algoriphagus marincola |
| 27 | A0A1G3QKY0 | 3e-148 | 49.7% | Predicted | Spirochaetes | Spirochaetes bacterium RBG_16_49_21 |
| 28 | A0A142EMH7 | 4.8e-147 | 52.1% | Predicted | Bacteroidetes | Algoriphagus sp. M8-2 |
| 29 | F8E8X6 | 4.3e-147 | 50.9% | Predicted | Deferribacteres | Flexistipes sinusarabici |
| 30 | B3QXC0 | 1.4e-146 | 50.0% | Predicted | Chlorobi | Chloroherpeton thalassium |
| 31 | A0A1G3QF30 | 1.4e-146 | 48.5% | Predicted | Spirochaetes | Spirochaetes bacterium RBG_13_51_14 |
| 32 | A0A1H5X473 | 7.7e-146 | 51.4% | Predicted | Bacteroidetes | Algoriphagus boritolerans |
| 33 | A0A150XI76 | 4.1e-144 | 51.2% | Predicted | Bacteroidetes | Roseivirga spongicola |
| 34 | A0A162PEH6 | 4.3e-144 | 50.9% | Predicted | Bacteroidetes | Flavihumibacter sp. CACIAM 22H1 |
| 35 | A0A1H1LV94 | 7e-144 | 51.9% | Predicted | Bacteroidetes | Gillisia sp. Hel1_33_143 |
| 36 | A0A090W8W5 | 9.1e-144 | 51.5% | Predicted | Bacteroidetes | Jejuia pallidilutea |
| 37 | G8TCM8 | 1.6e-143 | 51.1% | Predicted | Bacteroidetes | Niastella koreensis |
| 38 | F2LU51 | 1.1e-143 | 49.4% | Predicted | Proteobacteria | Hippea maritima |
| 39 | F6GCK3 | 2.6e-143 | 51.0% | Predicted | Bacteroidetes | Lacinutrix sp. (strain 5H-3-7-4) |
| 40 | F0SSC3 | 1.2e-142 | 49.7% | Predicted | Planctomycetes | Rubinisphaera brasiliensis |
| 41 | L1PJ68 | 4.7e-143 | 49.6% | Predicted | Bacteroidetes | Capnocytophaga sp. oral taxon 326 |
| 42 | E4MNR3 | 9.4e-143 | 50.4% | Predicted | Bacteroidetes | Capnocytophaga ochracea |
| 43 | A3XNM4 | 1.4e-142 | 51.1% | Predicted | Bacteroidetes | Leeuwenhoekiella blandensis |
| 44 | J1GPW6 | 2.7e-142 | 50.4% | Predicted | Bacteroidetes | Capnocytophaga sp. oral taxon 335 |
| 45 | C7M6E3 | 2.7e-142 | 50.4% | Predicted | Bacteroidetes | Capnocytophaga ochracea |
| 46 | I8UN96 | 5.3e-142 | 50.2% | Predicted | Bacteroidetes | Capnocytophaga sp. oral taxon 412 |
| 47 | L1NSS4 | 5.3e-142 | 50.2% | Predicted | Bacteroidetes | Capnocytophaga sp. oral taxon 380 |
| 48 | L1P559 | 5.3e-142 | 50.0% | Predicted | Bacteroidetes | Capnocytophaga sp. oral taxon 324 |
| 49 | A0A1E4H734 | 7.6e-142 | 50.6% | Predicted | Bacteroidetes | Sphingobacteriales bacterium SCN 48-20 |
| 50 | A0A0C1K207 | 9.9e-142 | 50.7% | Predicted | Chlamydiae | Neochlamydia sp. TUME1 |
| 51 | A0A0C5WBE4 | 7.5e-142 | 50.3% | Predicted | Bacteroidetes | Siansivirga zeaxanthinifaciens |
| 52 | S3BN00 | 7.5e-142 | 50.2% | Predicted | Bacteroidetes | Capnocytophaga sp. oral taxon 336 |
| 53 | A0A0C1JM08 | 1.4e-141 | 50.7% | Predicted | Chlamydiae | Neochlamydia sp. EPS4 |
| 54 | A0A090WEC3 | 1.7e-141 | 49.9% | Predicted | Bacteroidetes | Nonlabens ulvanivorans |
| 55 | A0A142H979 | 4.1e-141 | 50.2% | Predicted | Bacteroidetes | Hymenobacter sp. PAMC 26554 |
| 56 | A0A0M4H340 | 2.1e-141 | 50.2% | Predicted | Bacteroidetes | Capnocytophaga sp. oral taxon 323 |
| 57 | A0A084JYT1 | 3.3e-141 | 49.7% | Predicted | Bacteroidetes | Nonlabens ulvanivorans |
| 58 | A0A150X0N7 | 3.9e-141 | 49.9% | Predicted | Bacteroidetes | Roseivirga ehrenbergii |
| 59 | A0A1G0PCU1 | 8.1e-141 | 48.5% | Predicted | Ignavibacteriae | Ignavibacteria bacterium GWB2_35_6b |
| 60 | F4AW19 | 1e-140 | 50.8% | Predicted | Bacteroidetes | Dokdonia sp. (strain 4H-3-7-5) (Krokinobacter sp. (strain 4H-3-7-5)) |
| 61 | Q26BD2 | 1.3e-140 | 49.9% | Predicted | Bacteroidetes | Flavobacteria bacterium (strain BBFL7) |
| 62 | A0A081DC23 | 1.9e-140 | 49.9% | Predicted | Bacteroidetes | Nonlabens ulvanivorans |
| 63 | A0A1H5XN61 | 2.4e-140 | 50.4% | Predicted | Bacteroidetes | Chryseobacterium humi |
| 64 | A0A090PKG7 | 2.7e-140 | 49.9% | Predicted | Bacteroidetes | Nonlabens ulvanivorans |
| 65 | F8C318 | 2.2e-140 | 49.9% | Predicted | Thermodesulfobacteria | Thermodesulfobacterium geofontis |
| 66 | A3J1L4 | 3.4e-140 | 50.0% | Predicted | Bacteroidetes | Flavobacteria bacterium BAL38 |
| 67 | A0A1C2GLC0 | 4.6e-140 | 50.5% | Predicted | Bacteroidetes | Mucilaginibacter sp. PPCGB 2223 |
| 68 | A0A091G069 | 3.2e-140 | 51.2% | Predicted | Proteobacteria | Smithella sp. SCADC |
| 69 | A0A0A6Y4X8 | 4.7e-140 | 49.3% | Predicted | Bacteroidetes | Capnocytophaga sp. oral taxon 329 |
| 70 | A0A1J5EU80 | 4.8e-140 | 49.7% | Predicted | Bacteroidetes | Flavobacteriaceae bacterium CG2_30_31_66 |
| 71 | I3ZX55 | 6.9e-140 | 50.1% | Predicted | Bacteroidetes | Ornithobacterium rhinotracheale |
| 72 | A1ZNI8 | 9.5e-140 | 50.1% | Predicted | Bacteroidetes | Microscilla marina |
| 73 | A0A1J5KVH6 | 1.5e-139 | 49.4% | Predicted | Bacteroidetes | Lacinutrix sp. MedPE-SW |
| 74 | E2N3L2 | 2.9e-139 | 48.9% | Predicted | Bacteroidetes | Capnocytophaga sputigena |
| 75 | A0A0C1IBC8 | 6.5e-139 | 48.7% | Predicted | Bacteroidetes | Flavihumibacter sp. ZG627 |
| 76 | D4H214 | 4.2e-139 | 48.9% | Predicted | Deferribacteres | Denitrovibrio acetiphilus |
| 77 | A0A0X8JCC9 | 1.2e-138 | 48.3% | Predicted | Bacteroidetes | Capnocytophaga haemolytica |
| 78 | A0A0K8R150 | 2.1e-138 | 48.9% | Predicted | Bacteroidetes | Bacteroidales bacterium 6E |
| 79 | A0A1F9M1Z3 | 2.6e-138 | 49.3% | Predicted | Proteobacteria | Deltaproteobacteria bacterium RIFOXYA12_FULL_61_11 |
| 80 | I3C1W0 | 5e-138 | 49.0% | Predicted | Bacteroidetes | Joostella marina |
| 81 | G0J6E4 | 9.2e-138 | 47.8% | Predicted | Bacteroidetes | Cyclobacterium marinum |
| 82 | A0A0E9MXI8 | 1.3e-137 | 50.1% | Predicted | Bacteroidetes | Flavihumibacter petaseus |
| 83 | D3P8N7 | 1.8e-137 | 49.5% | Predicted | Deferribacteres | Deferribacter desulfuricans |
| 84 | A0A0D0F408 | 1.9e-137 | 49.4% | Predicted | Bacteroidetes | Flavobacterium hibernum |
| 85 | H1Y4A8 | 3.3e-137 | 49.3% | Predicted | Bacteroidetes | Mucilaginibacter paludis |
| 86 | A0A135L2J9 | 5.5e-137 | 49.8% | Predicted | Firmicutes | Tepidibacillus decaturensis |
| 87 | L1PAC4 | 1.1e-136 | 48.2% | Predicted | Bacteroidetes | Capnocytophaga sp. oral taxon 332 |
| 88 | A0A1B6A497 | 1.6e-136 | 49.5% | Predicted | Firmicutes | Tepidibacillus sp. HK-1 |
| 89 | A0A167UL70 | 6e-136 | 48.1% | Predicted | Bacteroidetes | Flavobacterium fryxellicola |
| 90 | A0A0H5DNR8 | 5e-135 | 49.5% | Predicted | Chlamydiae | Estrella lausannensis |
| 91 | A0A1C3FEX5 | 4.2e-135 | 48.1% | Predicted | Nitrospirae | Thermodesulfovibrio sp. N1 |
| 92 | A0A1G0WNE6 | 7.3e-135 | 49.4% | Predicted | Ignavibacteriae | Ignavibacteria bacterium RIFOXYC2_FULL_35_21 |
| 93 | G0L7L9 | 1.3e-134 | 47.8% | Predicted | Bacteroidetes | Zobellia galactanivorans (strain DSM 12802) |
| 94 | A0A0U5JEJ7 | 1.5e-134 | 47.8% | Predicted | Chlamydiae | Protochlamydia naegleriophila |
| 95 | D6YVJ1 | 1.8e-134 | 47.4% | Predicted | Chlamydiae | Waddlia chondrophila |
| 96 | F8LCE1 | 1.8e-134 | 47.4% | Predicted | Chlamydiae | Waddlia chondrophila |
| 97 | M1E5J8 | 1.8e-134 | 47.1% | Predicted | Firmicutes | Thermodesulfobium narugense |
| 98 | A0A1F9D273 | 2.4e-134 | 48.0% | Predicted | Proteobacteria | Deltaproteobacteria bacterium RBG_19FT_COMBO_43_11 |
| 99 | A0A1E5L380 | 4.4e-134 | 47.8% | Predicted | Firmicutes | Firmicutes bacterium MLFW-2 |
| 100 | F8KYM2 | 4.7e-134 | 48.2% | Predicted | Chlamydiae | Parachlamydia acanthamoebae |

**Table S4.** Number of proteins identified after LC/MS-MS analysis.

Experiment was performed at three biological replicas at each of the growth conditions.

| **Sample** | **Biological replica** | **Number of proteins** | **Number of peptides** | **Number of MS/MS spectra** | **FDR, %** |
| --- | --- | --- | --- | --- | --- |
| Maltose fermentation | 1 | 932 | 5220 | 34903 | 0.49 |
|  | 2 | 802 | 4347 | 33959 | 0.56 |
|  | 3 | 912 | 5289 | 32896 | 0.57 |
| Aerobic respiration  on acetate | 1 | 724 | 4082 | 32631 | 0.85 |
|  | 2 | 769 | 4341 | 33130 | 0.83 |
|  | 3 | 724 | 4103 | 32414 | 0.67 |

**Table S5** (presented as a separate MS Excel file).

Results of peptides identification.

**Table S6** (presented as a separate MS Excel file).

Results of proteins identification.

**Supplementary Figure S1.** Relative transcript abundance of *atpA* versus *rpoB* reference housekeeping genes of *M. roseus* grown at different growth conditions. Note the mutual overlay of error bands at all the growth conditions.


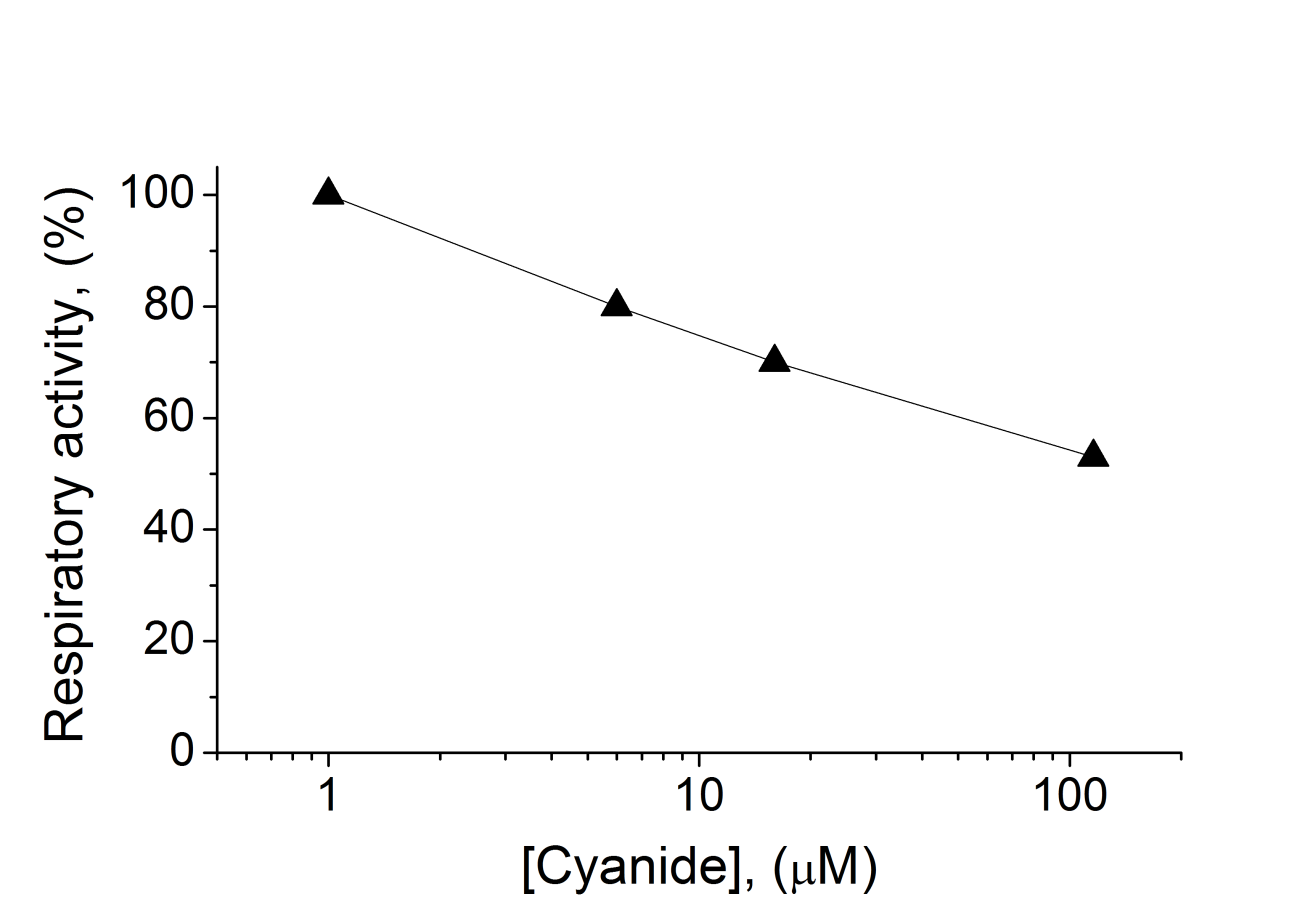


**Supplementary Figure S2.** The effect of cyanide on aerobic respiration of *M. roseus* resting cells with acetate as the electron donor (graph is shown in logarithmic scale). The respiratory activity in the absence of cyanide reached 1.9 nmol O_2_ min^-1^ mg^-1^ of cell protein and was defined as 100%. The experiment was performed in triplicate, mean values are presented.


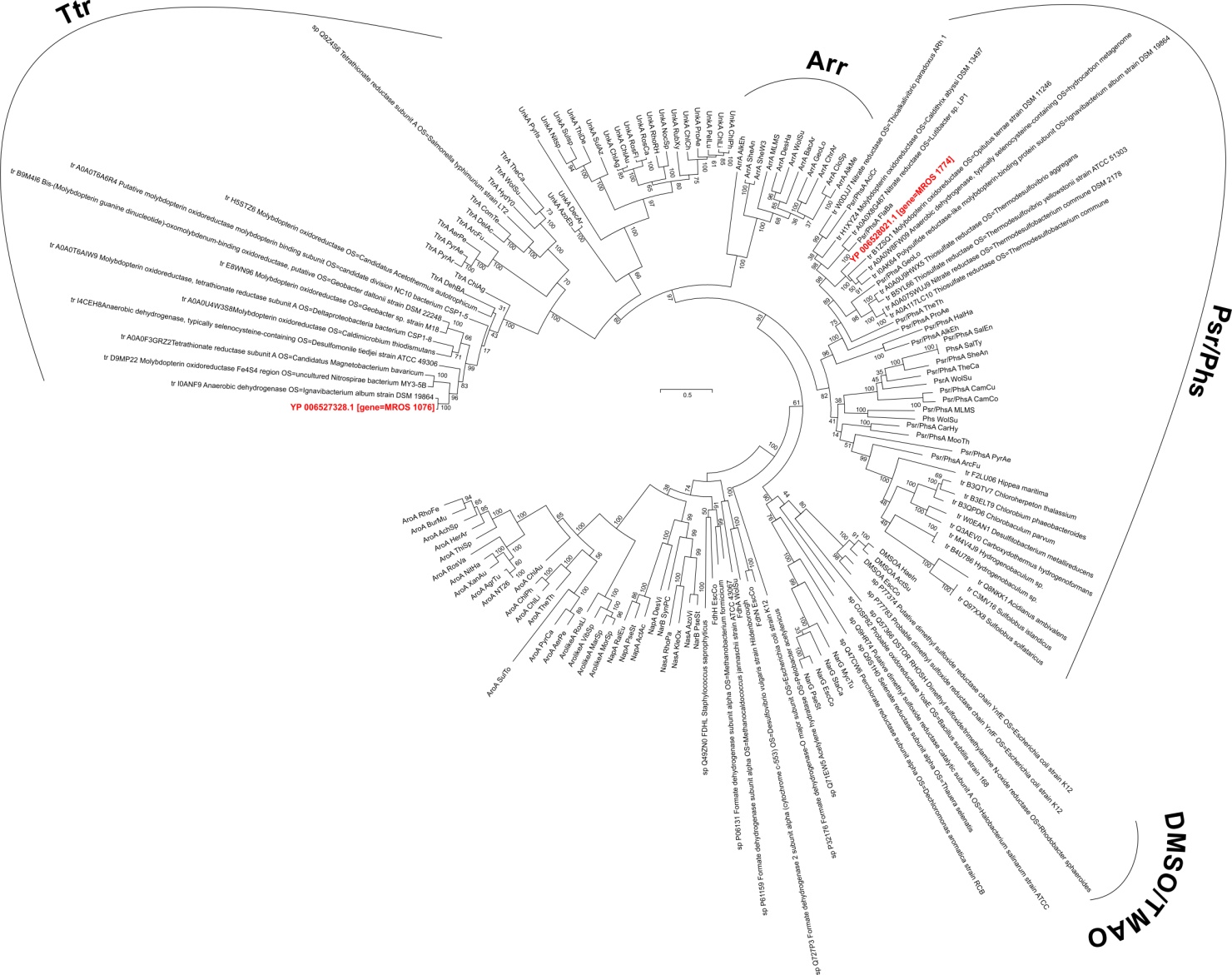


**Supplementary Figure S3.** Maximum Likelihood phylogenetic tree of CISM oxidoreductase catalytic subunits A. A total of 148 sequences were involved in the analysis. The tree with the highest log likelihood (-106928.6626) is shown. The bootstrap values (100 replicates) are shown next to the branches. All positions with less than 95% site coverage were eliminated. There were a total of 539 positions in the final dataset. The tree was constructed in MEGA6 (Tamura et al., 2013). Two *M. roseus* proteins (MROS_1076 and MROS_1774) are in red. Ttr – tetrathionate reductase family; Psr/Phs – polysulfide/thiosulfate reductase family; Arr – arsenate reductase family; DMSO/TMAO – dimethyl sulfoxide/trimethylamine *N*-oxide reductase family. Previously described non-Arr-type arsenate reductases of *Pyrobaculum aerophilum* (Cozen et al. 2009) – TtrA PyrAe (tetrathionate reductase) and Psr/PhsA PyrAe (polysulfide/thiosulfate reductase). Bar is 0.5 substitutions per site.
